# Professional development among newly graduated registered nurses working in acute care hospital settings: A qualitative explorative study

**DOI:** 10.1111/jonm.13771

**Published:** 2022-09-02

**Authors:** Anna Willman, Jan Nilsson, Kaisa Bjuresäter

**Affiliations:** ^1^ Department of Health Sciences, Faculty of Health, Science, and Technology Karlstad University Karlstad Sweden; ^2^ Department of Health and Social Sciences University of Innlandet Elverum Norway

**Keywords:** acute care hospital, leading nursing care, newly graduated registered nurses, qualitative design

## Abstract

**Aim:**

To explore newly graduated registered nurses' perceptions of their work situation and management of nursing care in complex patient situations after 18 months of work experience.

**Background:**

Newly graduated registered nurses working in acute care hospital settings play a critical role in providing safe nursing care.

**Methods:**

An explorative qualitative design, with four focus group interviews with 14 newly graduated registered nurses working in acute care hospital settings.

**Results:**

One theme emerged: ‘Clarity and security in one's own nursing role despite facing challenges that hinder professional development’ and three categories: ‘Independency due to one's own efforts and experience’, ‘Well‐functioning teamwork’ and ‘Challenges in the work situation’.

**Conclusion:**

After 18 months in the profession, the nurses were considered to be advanced beginners; at the same time, the most experienced nurses on their respective wards. They found it challenging and need to further develop competences concerning managing and organizing the nursing care of several complex patient situations or new patient groups, as well as supervising novice registered nurses and nursing students.

**Implication for Nursing Management:**

Powerful and urgent action is needed to be taken by national healthcare policymakers as well a hospital and nurse managers to develop long‐term strategies to improve working conditions for newly registered graduated nurses.

## 1 INTRODUCTION

Today's newly graduated registered nurses (NGRNs) who commence work in acute care hospital settings will face fast‐changing healthcare systems that are characterized by an increasing number of patients with acute, chronic and complex co‐morbidities (Hussein et al., 2017), shorter hospital lengths of stay (Buchan et al., 2013) and a shortage of registered nurses (RNs) (WHO, 2020c). This can lead to higher demands being placed on NGRNs who are expected to provide comprehensive nursing care that meets patients' complex and diverse needs in acute care hospital settings (Bandini et al., 2018).

## 2 BACKGROUND

RNs' competence is developed by gaining experience over time in conjunction with gaining theoretical knowledge and the ability to be reflective. RNs describe themselves being motivated to pursue professional development as it is a way to increase competence, comply with requirements, deepen knowledge and increase career possibilities (Falk & Lindström, 2022; Pool et al., 2016). For further professional development, teamwork (Eddy et al., 2016; Serafin et al., 2022) and leadership that supports teamwork and facilitates professional development are of importance (Page et al., 2020). WHO (2020a; 2020b) is also arguing for the importance of RNs' competence concerning leadership, which must be highlighted at all levels in healthcare organizations.

In recent years, acute care hospital settings have become increasingly specialized and complex (Disch et al., 2016), primarily due to the increasing number of patients who are in need of more demanding and complex nursing care in acute care hospitals (Carnesten et al., 2022; Dharmaraijan et al., 2016). Along with the heightened complexity of patient cases admitted to hospitals, there has also been a constant shortage of RNs (ICN, 2019), in particular of experienced RNs (National Board of Health and Welfare, 2018). Organizing healthcare without a sufficient number of RNs may lead to a decline in patient safety (Aiken et al., 2017; Driscoll et al., 2018), and patients in complex situations have been found to have a higher risk of mortality and lower probability of home discharge (Bandini et al., 2018).

Nursing care in complex patient situations are dynamic interactions related to instability, variability and uncertainty from the perspective of the patient and the RNs with processes such as personal, communication and cognitive, psychosocial and ethical aspects interacting with each other causing uncertainty and unpredictability in a situation (Huber et al., 2020; Kannampallil et al., 2011). In order to handle nursing care in complex patient situations successfully, RNs need professional experience, expertise and clinical reasoning skills, as well as contextual prerequisites such as time resources and support through teamwork (Kentischer et al., 2018). Previous studies on NGRNs have focused on their transition from education into professional life and describe them facing new demands and challenges in complex patient situations (Arrowsmith et al., 2016; Cheng et al., 2021; Walton et al., 2018) and that both organizational and personal factors influence their professional development (Charette et al., 2019). However, there is limited knowledge regarding NGRNs' perceptions of their working situation, how they manage nursing care in complex patient situations in demanding and complex acute care hospital settings after their first one and a half year in the profession. What is the understanding of NGRNs about their work situation in nursing care in an acute care hospital? How does the careful management of inpatient nursing care for these patients occur? Therefore, the aim of the study was to explore NGRNs' perceptions of their work situation and management of nursing care in complex patient situations after 18 months of work experience.

## 3 METHODS

An explorative qualitative design was used to gain a deeper understanding of a predetermined subject (Polit & Beck, 2021), and an inductive manifest and latent content analysis was performed as described by Krippendorff (2018). Data were collected through focus group interviews (FGIs) (Morgan, 1997). FGIs aim to access a deeper understanding of a predetermined subject as they allow participants to interact with each other and explore their perceptions, views and opinions using their one's own words (Morgan, 1997). In accordance to EQUATOR Network, the COREQ criteria for reporting was used (Data S1) (Tong et al., 2007).

### 3.1 Context

Study participants consisted of NGRNs who were employed by a county council in central Sweden. The NGRNs worked in direct patient care in a central regional acute care hospital on a variety of wards including medical, surgical, emergency, gynaecological, psychiatric and oncological wards. In this study, the NGRNs are considered to be advanced beginners, due to RNs considering themselves to be novices or advanced beginners for their first 2 years of professional life (Benner, 2001).

### 3.2 Sample and recruitment

The NGRNs who participated in this study were recruited using convenience sampling (Polit & Beck, 2021). They received oral and written information about the study at the beginning of their clinical development programme. The first author was assisted by a ward head nurse to gain access to eligible NGRNs who had 18 months of clinical experience. The first author contacted the participating NGRNs using telephone text messages to arrange the time and place for the FGIs. Prior to the FGIs, interested NGRNs received an information letter and provided written informed consent. In total, 14 NGRNs agreed to participate in the study. Their ages ranged between 24 and 30 (mean age 26.8 years), and 12 participants were women and two were men.

### 3.3 Ethical considerations

Ethical considerations were made in accordance to the Helsinki Declaration (2013). Before each interview, informed consent was collected. They were also informed that participation was voluntary and that they were free to end their participation at any time without giving an explanation. The study was given ethical approval by the National Ethical Review Board.

### 3.4 Data collection

As a point of departure, each FGI started off with a question asking participants to describe their understanding of complex patient situations related to instability, variability and uncertainty. The FGIs were conducted as semi‐structured discussions and were based on a semi‐structured interview guide (Table 1). In addition to the semi‐structured interview guide, follow‐up questions were asked. They lasted between 43 and 62 min (mean 56 min) and were recorded and transcribed verbatim.

**TABLE 1:** Interview guide for the semi‐structured focus group interviews

Opening question	Can you please tell us how you perceive your work situation?
Transition question	Can you describe a patient situation that you perceived as complex?
Key questions	Can you describe how you perceive and manage nursing care in complex patient situations?
	Can you describe a work situation in a complex patient situation that worked well?
	Can you describe a work situation in a complex patient situation that did not work well?
Closing question	Is there anything else you would like to add?

### 3.5 Data analysis

The transcribed data were analysed using a text‐driven, interpretive qualitative manifest and latent content analysis that included several steps (Krippendorff, 2018). The interviews were firstly read several times to get an overall understanding of the data. Thereafter, they were read thoroughly to identify manifest meaning units, which were used to formulate codes. At this stage of the analysis, data are based on manifest content that clearly emerge from the transcribed text. The codes were then grouped into subcategories based on similarities and differences. The contents in the subcategories were interpreted and sorted into latent categories. As the subcategories were built on manifest data, then more complex analyses and interpretations were made following the methodological process of going back and forth in the text to find different levels of abstraction to increase the ability to see context and patterns (Krippendorff, 2018). The authors then agreed on eight subcategories that the three categories are based upon, which then emerge in one overarching theme (Table 2). The last step of the process was to confirm the relevance of the results by verifying the correlation with the aim and the categories and the overarching theme (Krippendorff, 2018).

**TABLE 2:** Examples of the analysis process

Meaning units	Codes	Categories	Overachieving theme
This unforeseen situation happens quite often … you never know what will happen, various complications and consequences arise that you should try to deal with … it is not as stressful as before, you are calmer and more confident (FGI 2)	Calmer and more confident in unforeseen situations	Independency due to one's own efforts and experience	Clarity and security in one's own nursing role despite facing challenges that hinder professional development
The team that joins in and solves difficult patient situations together … afterwards you need to help out because you have lots of patients who have not received care and it is their turn (FGI 3)	Teamwork solving difficult situations and helping each other	Well‐functioning teamwork	
It's going ok but when you have lots of really sick patients and you have to help the newer nurses it is hard to have time to do everything you have to do and then you feel like a failure (FGI 1)	Hard to have time to help novice nurses when there are lots of really sick patients	Challenges in the work situation	

## 4 RESULTS

One overarching theme ‘Clarity and security in one's own nursing role despite facing challenges that hinder professional development’ and three categories emerged: ‘Independency due to one's own efforts and experience’, ‘Well‐functioning teamwork’ and ‘Challenges in the work situation’ (Table 3). The NGRNs perceived that they gained competence, independency and confidence in the nursing care of complex patient situations they had experience of, resulting in clarity and security in their role as an NGRN. They managed complex patient situations thought leading and collaborating with colleagues and patients. They found that facing challenges in the work situation reduced their ability to manage the nursing care of several complex patient situations simultaneously or complex patient situations they are not familiar with, such as patients who usually stay on other wards. These challenges were also perceived to be a hinder in their professional development.

**TABLE 3:** Subcategories, categories and the overarching theme

Subcategories	Categories	Overarching theme
Competence gained through experience and their one's own efforts	Independency due to one's own efforts and experience	Clarity and security in one's own nursing role despite facing challenges that hinder professional development
Confidence and independency in the role		
Teamwork	Well‐functioning teamwork	
Leading and delegating care		
Advocates for patients' needs		
Involving patients in their one's own care		
Given the responsibilities of an experienced RN	Challenges in the work situation	
Supervising novice RNs and nursing students		

### 4.1 Independency due to one's own efforts and experience

After being in the profession for 18 months, the NGRN had gained experience of nursing in complex patient situations. Due to *competence gained through experience and their one's own efforts* along with struggles, they perceived that their professional competence had increased and that they had gained *confidence and independency in the role as an RN*. A common opinion was that experience gained through working as an RN along with a gained confidence and positive attitude had contributed to this and developed the ability to provide optimal nursing care in complex patient situations. They had also learnt how to prioritize different tasks and develop routines for tasks that they carried out repeatedly, and they had gained experience through handling several different complex patient situations in the acute care hospital settings they worked in.

… there was a patient who had a troublesome venous port and he did not want me to use it because it has bothered him so much, I said we should try … I think he felt I knew what I was doing and then it wasn't a problem, it probably has a lot to do with the attitude you put across …. I also feel that if you have better self‐confidence, then things will work out well …. (FGI 1, P 2, P 4)

The NGRNs had gained a sense a confidence in their role as an RN and regarded nursing in complex patient situations. This also led to feeling safe and competent when administrating medication in complex patient situations.

… the days go by easier and you have more time to check medication that you do not know about and the margins are better for doing a better job … (mmm yes I agree). … now you can see what needs to be done straight away and what can wait. (FGI 4, P 2, P3)

Experience in being able to identify a patient's deteriorating condition came from taking the approach of remaining calm when assessing situations as critical. Being confident and secure in their profession meant that they knew how they would act and react and that they had the skills required to manage complex patient situations. This has been done due to experience and one's own struggles meaning despite the fact that they were previously afraid, they have continued to expose themselves to new situations.

… I feel that I am not as afraid of new situations anymore—I have a better basic knowledge and I can judge if a situation is critical and I know what is critical and what is not. (FGI 1, P 1)

### 4.2 Well‐functioning teamwork

Regarding nursing care in complex patient situations, the participating NGRNs described effective collaboration between nurses, assistant nurses and doctors as being of importance. When the NGRNs experienced a feeling of *teamwork* that worked well with assistant nurses and doctors, and to manage complex patient situations more effectively, they were then able to complement each other by sharing their different experiences and knowledge. The NGRNs also described that having a team that worked well together was more important than having an experienced RN available as a mentor. The members of the team complimented each other with their different experiences and together there was a feeling of ‘we’.

When we are all working together, we can help each other if needed and share each other's experiences and knowledge. (FGI 3, P 2)

NGRNs described how they managed complex patient situations by *leading and delegating care* in these situations. They expressed that they had grown into the leadership role and felt more secure by gaining an overall understanding of how nursing work is to be performed. They also described how they could be determined and driven when working alongside colleagues and follow up and monitor how assistant nurses performed their work. Various stages in the nursing process in complex nursing situations could be delegated to colleagues to relieve their one's own workloads. However, despite the NGRNs saying they were comfortable in their leadership roles, they felt that further development was necessary in this area.

After the rounds I have to priorities and make decisions … you have to remember that you have assistant nurses … you need to take on the leadership role and say that I need to do this and you need to do that, etc … you have to make a plan and delegate … the assistant nurses want us to take the role of the leader. (FGI 2, P 4)

In communication with colleagues, the NGRNs *advocate for patients' needs*. Communication between colleagues was described as important in order to manage and give patients the correct nursing care in complex patient situations. The NGRNs expressed that they could participate in these conversations on equal terms with the doctors and other colleagues and that they showed courage and responsibility in these conversations by questioning or expressing their views to meet patients' needs on how nursing care should be performed. They described the importance of all team members having updated information about patients, and they strived to share information with each other even though it was stressful.

… I can be decisive in my role as a nurse and I can say that I have a plan for this patient … I can watch out for the patient … I'm tougher now …. (FGI 1, P 3)

Participating NGRNs managed nursing care by *involving patients in their one's own care*; this was perceived as vital to include in conversations, tasks and the exchange of information with the patient. Providing patients with more information gave them the opportunity to participate in their one's own care, which was considered important because this group of patients was often seriously ill.

If patients are informed and we know what they want and what they are striving for … they become more motivated … it's good that they are aware … so they get the best care. (FGI 4, P 1)

Communication also included relatives who, with information and knowledge about care plans, became more familiar with the patient's care and felt involved and calm. Having experience and knowledge of communicating with both the patient and their relatives was thought to result in a relaxed relationship between the NGRNs, the patient and their relatives, where trust in the nurse was shown.

### 4.3 Challenges in the work situation

Participating NGRNs perceived that their work situations were sometimes challenging as they perceived that they were *given the responsibilities as an experienced RN* and asked to *supervise novice RNs and nursing students*. These work situations and their conditions made managing nursing care in complex patient situations more difficult. It emerged that the NGRNs, despite only having 18 months of work experience, were now among the RNs who had the most experience on their wards, which also meant that they were expected to work extra shifts due to experienced RNs being required. The lack of experienced RNs resulted in them being afraid of becoming overworked and burnt out. The high nurse turnover led to experienced RNs who quit being replaced by novice RNs and also led to experience and knowledge disappearing from the ward, making the NGRNs work situations more difficult.

We have had a lot of novice nurses … there have been nine new ones … it was a real transition to get experienced … (another nurse continues) It takes almost a year to do it … there are not many experienced ones anymore … there are 11 nurses who have worked 18 months or less … a lot of knowledge is disappearing. (FGI 3, P 1,P 3)

Responsibility for providing nursing care in several complex patient situations simultaneously or the responsibility for providing nursing care to patients from other wards was perceived as demanding. Managing the nursing care of several complex patient situations involved assessing which patients had the greatest needs. When participating NGRNs cared for patients from other departments and patient groups that do not normally come to their ward, they found that their competence was challenged. In these situations, they did not feel they had the competence to prioritize and delegate work to assistant nurses due to being responsible for providing nursing care to several complex patient situations simultaneously. Thus, these patients' needs are unfamiliar to the NGRNs, which leads to both themselves and the patients feeling insecure.

The patients become unsure … we are unsure about what we are doing … then they get worried … and we might do the wrong thing if we aren't so experienced in, for example, head injuries … (you have more of this, addressing another NGRN) … things can go wrong where these patients are concerned …. (FGI 1, P 3)

Challenges of a pedagogical nature, such as supervising novice RNs and nursing students, made them feel insufficient when nursing in complex patient situations. Supervising novice RNs in complex patient situations when they themselves needed support from an experienced RN was described as challenging. They found it difficult to coordinate nursing in complex patient situations when there were a large number of novice RNs and newly graduated doctors. It appeared that even newly graduated doctors would turn to the NGRNs with 18 months of experience for help regarding routines and expect them to make decisions regarding care in complex patient situations. The NGRNs expressed having insufficient competence to be responsible for patients in complex situations as well as recently graduated doctors and novice RNs—the pressure of these situations led to an increase in stress levels and perceived pressure.

… when you have lots of really sick patients and you have to help colleagues who are less experienced, … you feel like there is not enough of you to go round … now you have to make judgments yourself, if there were more experienced nurses you would have asked … now you have to make the decision yourself. (FGI 2, P3)

## 5 DISCUSSION

The aim of this study was to explore NGRNs' perceptions of their work situation and management of nursing care in complex patient situations after 18 months of clinical experience. Through experience gained from nursing care in complex patient situations on the wards, the NGRNs who participated in this study have achieved clarity, autonomy and have gained confidence in their role as RNs. They manage nursing care in complex patient situations by leading, delegating and collaborating with the team. However, in this study, the NGRNs were the most experienced RNs after just 18 months of clinical experience. Further, they were expected to be responsible of the nursing care of several complex patient situations or new patient groups and supervising novice RNs and student nurses. This findings has not been shown elsewhere to our knowledge, and for the NGRNs, this working situation is very worrying and utterly remarkable. In contrast, Benner (2001) is referring to RNs with 18 months in the profession as advanced beginners. As a consequence identified in the present study is that where there is high staff turnover of RNs, knowledge disappears from the wards and this is a threat to RNs' professional development. Earlier research has shown that a lack of peer support is a barrier to professional development (Page et al., 2020). Organizational support and strong nursing leaders play a vital role in RNs' professional development, for example, by moving away from traditional hierarchies and identifying individual RNs' clinical competence and need for further training (King et al., 2021). However, this presupposes that the manager has genuine knowledge in evidence base nursing care with the adequate level of education in order to be able to make right assessments and support each individual NGRN after 18 months within the profession.

These results reflect the consequences of the global nursing shortage (Buchan et al., 2013), which is estimated to increase in the coming years, both in Sweden (National Board of Health and Welfare, 2018) and other countries (ICN, 2019). The nursing shortage is a threat to sustainable health systems, which in turn will have an impact on reaching the goal of health for all by 2030 (WHO, 2018). As a response to the global nursing shortage, WHO (2020c) advocates for a massive acceleration in the education of new RNs to bridge the gap of six million nurses and midwives. It is therefore of importance to focus on RNs' working conditions and keeping them in the profession as this shortage may not be able to be solved merely by educating more RNs as recommended by (WHO, 2020c).

The results of the present study show that one obstacle in achieving professional development among NGRNs was the uncertainty associated with supervising NGRNs with less experience in complex patient situations. Other studies have reported that novice RNs report low levels of competence in supervising students and staff (Gardulf et al., 2019; Nilsson et al., 2019) and in the organization of nursing care (Halabi et al., 2020). One possible way to support the transition and professional development in working in the context of acute care hospital settings among NGRNs has been shown to be peer learning (Pålsson et al., 2018). It is therefore of importance for ward managers to utilize research evidence when organizing teams in terms of RNs with different experience levels of overall competence in the health care team as recommended by the National Board of Health and Welfare (2015). Well‐composed teams could increase RNs' job satisfaction and thus increase the chances of retention. It is the employer's responsibility to provide RNs with the conditions required to be able to carry out nursing care with high patient safety and good quality, as these are important predictors for wanting to remain in the profession.

Previous research shows that even RNs with approximately 5 years of experience find nursing in complex situations challenging or overwhelming (Kentischer et al., 2018). The participating NGRNs in the present study had the ability to manage the nursing care of single complex patient situations, but when presented with several cases simultaneously or complex patient situations they not were familiar with, they found it more difficult. The difficulties were related to managing the making of assessments and prioritizing and organizing care, and the NGRNs felt that they took a step back in their professional development as a consequence of these difficulties. It has been found that nursing in complex patient situations can lead to incomplete care or the avoidance of providing care, which can be a threat to patient safety, especially when under time pressure (Vinckx et al., 2018). To accomplish an effective nursing process in complex patient situations and the best outcome for the patient, the level of the RN's attention, knowledge and experience is crucial (Huber et al., 2020) as shown in this study among NGRNs' nursing care in single complex patient situations. Another strong incitement for NGRNs not being given sole responsibility for several complex patient situations simultaneously is that an association between the complexity of nursing care and turnover among NGRNs has been found (Ten Hoeve et al., 2020). Hence, it is not appropriate to make NGRNs responsible for leading a team, assessing, prioritizing and organizing care for several patients in complex situations or unfamiliar cases usually treated on other wards when they have only been in the profession for 18 months, due to the risk of increased turnover and reduced patient safety.

### 5.1 Limitations

As suggested by Morgan (1997), a goal was to include FGIs with four to six participants; thus, this was not possible to organize. Despite there only were three participants in one FGI, they interacted well with each other and generated in richness of information as they shared their views, thoughts and opinions. FGIs aim to capture the interaction in the group to explore and discuss between the participants. The number of participants may also be seen as a limitation; however, saturation was discussed during the analysis process, and saturation was assessed to be reached after analysis of the fourth FGI. Using FGI was also a way to capture new knowledge. The head of the ward nurse recruited the participants; this could serve as a limitation as they might have asked the most positive NGRNs.

## 6 CONCLUSION

NGRNs with 18 months of professional experience are considered to be advanced beginners; however, due to high turnover in the profession, they are among the most experienced RNs on their respective wards. Despite having developed independency and clarity in their nursing role as well as leadership skills, they were facing challenges that made their work situation difficult. This was due to managing and organizing the nursing care of several complex patient situations or new patient groups, and supervising novice RNs and student nurses is challenging, and they need further support to develop competencies in these areas.

## 7 IMPLICATION FOR NURSING MANAGEMENT

Powerful and urgent action is needed at a national level by healthcare policymakers to develop long‐term strategies to improve NGRN's working conditions. Further, it is the responsibility of the healthcare leaders and management to improving work conditions and professional growth among all RNs to ensure that staffing is adequate, with a mix of different experiences and competences. Retaining the more experienced RNs within patient‐centred work the NGRNs would also benefit a security and role models. However, this presupposes that the manager has genuine knowledge in evidence base nursing care with the adequate level of education in order to be able to make right assessments and support each individual NGRN after 18 months with in the profession by developing long‐term strategies for improving their work condition and by establishing adequate staffing combinations by considering RNs' different experience levels that will contribute to the total competence within the team. Further, RNs need to be given a position in the healthcare systems, so they can have an impact of RNs work situation and facilitate a sustainable working life.

## CONFLICT OF INTEREST

The authors declare that they have no competing interests or conflict.

## ETHICS STATEMENT

The study followed the ethical principles in accordance with the Declaration of Helsinki (Helsinki Declaration, 2013) and was given ethical approval by the Ethical Review Board (reg. no. 2015/071 and 2015/071/2). Written informed consent was obtained from each participant in connection with the clinical development programme. Study participants received both oral and written information about the aim of the study, that their participation was voluntary and that they could end their participation at any time without explanation.

## Supporting information


**Data S1.** Supporting Information

## Data Availability

Data are available, but not for public due to ethics.
